# Cancer Cells with Alternative Lengthening of Telomeres Do Not Display a General Hypersensitivity to ATR Inhibition

**DOI:** 10.3389/fonc.2016.00186

**Published:** 2016-08-23

**Authors:** Katharina I. Deeg, Inn Chung, Caroline Bauer, Karsten Rippe

**Affiliations:** ^1^Research Group Genome Organization & Function, German Cancer Research Center (DKFZ) and Bioquant Center, Heidelberg, Germany

**Keywords:** alternative lengthening of telomeres, ataxia telangiectasia- and RAD3-related, ATR inhibitor, VE-821

## Abstract

Telomere maintenance is a hallmark of cancer as it provides cancer cells with cellular immortality. A significant fraction of tumors uses the alternative lengthening of telomeres (ALT) pathway to elongate their telomeres and to gain an unlimited proliferation potential. Since the ALT pathway is unique to cancer cells, it represents a potentially valuable, currently unexploited target for anti-cancer therapies. Recently, it was proposed that ALT renders cells hypersensitive to ataxia telangiectasia- and RAD3-related (ATR) protein inhibitors (Flynn et al., Science 347, 273). Here, we measured the response of various ALT- or telomerase-positive cell lines to the ATR inhibitor VE-821. In addition, we compared the effect of the inhibitor on cell viability in isogenic cell lines, in which ALT was active or suppressed. In these experiments, a general ATR inhibitor sensitivity of cells with ALT could not be confirmed. We rather propose that the observed variations in sensitivity reflect differences between cell lines that are unrelated to ALT.

## Introduction

Cancer cells need to maintain their telomeres to avoid cellular senescence and apoptosis induced by the replicative shortening of chromosome ends. Frequently, expression of TERT, the protein subunit of telomerase, is reactivated to extend the telomeres. In addition, a significant fraction of tumors elongates the telomeres by an alternative lengthening of telomeres (ALT) pathway that operates via DNA repair and recombination processes as reviewed previously (1, 2). ALT is not known to occur in healthy cells and, thus, represents a unique feature of cancer cells that could be targeted with specific drugs.

A recent study investigated telomerase-positive and ALT-positive osteosarcoma and lung cancer cell lines as well as glioma stem cell lines and reported that cells that employ the ALT pathway are hypersensitive to the inhibition of the protein kinase ataxia telangiectasia- and RAD3-related protein (ATR), one of the two main DNA damage checkpoint-activating kinases in human cells (3). The authors concluded that treatment with the ATR inhibitor VE-821 selectively kills ALT cells within 6 days. They proposed that the immediate cell death induced by ATR inhibition in ALT cells is caused by an accumulation of DNA damage, aberrant anaphase chromosome segregation, and increased micronuclei formation. Yet, how ATR inhibition elicits these effects specifically in ALT cells to affect the short-term cell viability remains elusive. Previously, several studies have demonstrated that inhibiting telomerase or ALT will induce senescence or cell death, e.g., Ref. (4–7). However, in the latter work many population doublings over weeks and months were required to elicit this type of response. This long-term response is in line with the view that no significant effects on cell viability due to telomere shortening are expected to occur within a few days.

Here, we recapitulated the cell viability and FACS experiments by Flynn et al. using various ALT- or telomerase-positive cell lines. Additionally, we investigated whether suppression of ALT activity affects cell viability upon treatment with ATR inhibitor. In our study, no general hypersensitivity of ALT-positive cells toward ATR inhibitors was observed.

## Materials and Methods

### Cell Culture

Validated U2OS, HeLa, CAL72, HCT116, and SAOS2 cells were obtained from the German Collection of Microorganisms and Cell Cultures (DSMZ, Braunschweig, Germany). The MG63 cell line was purchased from CLS Cell Lines Service (Eppelheim, Germany). U2OS, HeLa, and CAL72 cells were maintained in DMEM supplemented with 10% FCS, 2 mM l-glutamine, 1% antibiotics. For CAL72, 1X ITS liquid media supplement (Sigma-Aldrich) was added to this medium. The other three cell lines studied were cultured in 90% McCoy’s 5A supplemented with 10% FCS, 2 mM l-glutamine, and 1% antibiotics (HCT116), in 85% McCoy’s 5A supplemented with 20% FCS (SAOS2) or in DMEM/Ham’s F12 medium supplemented with 5% FCS, 2 mM l-glutamine, and 1% antibiotics (MG63). An U2OS cell line for inducible expression of ATRX (referred to here as U2OS^ATRX-2^) was kindly provided by Richard Gibbons (University of Oxford, UK) and has been described previously (8). The U2OS^ATRX-2^ cells were cultured in DMEM supplemented with 10% doxycycline-free FCS, 2 mM l-glutamine, 1% antibiotics, 0.5 μg/ml puromycin, 0.7 μg/ml G418.

### Cell Viability Assays

For cell viability assays, cells were seeded in triplicate in 96-well plates and incubated overnight. Different cell numbers were seeded to determine the cell number needed to obtain 70–90% confluency of the control sample after 6 days. Optimal starting cell numbers for U2OS, HeLa, HCT116, and MG63 were 500 cells, and for CAL72 and SAOS2 1500 cells. The following day cells were either treated with DMSO (control) or with increasing concentrations (0.5, 1, 2, 4, 8, and 16 μM) of the ATR inhibitor VE-821 dissolved in DMSO. Three different batches were compared that included VE-821 as a predissolved 10 mM solution or as a powder obtained from Selleckchem as well as VE-821 in powder form from Calbiochem/Merck. The three different VE-821 batches yielded undistinguishable dose–response curves when compared for the same reference cell samples. The compound from Selleckchem was used for the experiments presented here. Cells were incubated for 6 days without medium change and cell viability was analyzed using CellTiter Glo (Promega) and a TECAN Infinite M200 plate reader according to the manufacturers’ instructions.

### FACS Analysis of Cell Death

For analysis of cell death, cells were seeded in T25 flasks. Cell numbers were adjusted for each cell line to account for varying proliferation rates (1 × 10^5^ CAL72 cells, 1.4 × 10^5^ SAOS2 cells, and 0.8 × 10^5^ cells for HeLa, HCT116, and U2OS). The following day each cell line was either treated with 3 μM VE-821 (Selleckchem) or with the same volume of DMSO for the control samples. Cells were incubated for 6 days without medium change. Cells, including dead cells, were collected by trypsin and total cell numbers were determined using the LUNA cell counter (Biozym). Cells were resuspended in FACS binding buffer (10 mM HEPES, 2.5 mM CaCl_2_, 140 mM NaCl) at a final concentration of 2 × 10^6^ cells/ml, stained with FITC annexin V (BioLegend) and propidium iodide (Miltenyi Biotec) according to the manufacturers’ instructions, and analyzed by flow cytometry on a FACS Canto II (BD Biosciences). The fraction of apoptotic cells, characterized as annexin V positive, was quantified using the FACS Diva software. The percentage of induced cell death *d*_ind_ was calculated as

$$d_{\text{ind}}=\;\frac{\left[\left(\begin{array}{l} \text{percentage dead }\\ \text{cells treated} \end{array}\right)-\left(\begin{array}{l} \text{percentage dead }\\ \text{cells control} \end{array}\right)\right]}{\text{percentage viable cells control}}\times 100\%.$$

### Generation and Analysis of a U2OS Cell Line with Inducible ATRX Expression

The pEGFP-C2-ATRX-HA plasmid was kindly provided by David Picketts (University of Ottawa, Canada) (9). ATRX-HA was amplified and cloned into the pTRE3G-ZSGreen1 vector (Clontech, USA) to construct pTRE3G-ZSGreen1-ATRX-HA for doxycycline-inducible expression of ATRX. Plasmids that contained the ATRX cDNA were propagated in the *dam*/*dcm*-negative bacteria strain JM110 (Agilent Technologies) to avoid transposon insertions. A stable U2OS^ATRX-1^ cell line was generated by transfecting U2OS cells with pCMV-Tet3G (Clontech, USA) and pTRE3G-ZSGreen1-ATRX-HA and subsequent selection with G418 (1 mg/ml). U2OS^ATRX-1^ cells were cultured under the same conditions as the parental U2OS cell line except that doxycycline-free FCS was used. For induction of ATRX expression, 1 μg/ml doxycycline was added to the medium and the expression was evaluated by western blotting. The following antibodies were used: anti-ATRX (Sigma, HPA001906), anti-HA (Abcam, ab18181), and anti-GAPDH (Ambion, AM4300). ALT activity after ATRX expression was evaluated by the C-circle assay and the number of ALT-associated PML bodies (APBs) as described previously (10). U2OS^ATRX-1^ (−) and U2OS^ATRX-1^ (+) cells were cultured in the absence or presence of 1 μg/ml doxycycline, respectively, for at least 7 days before treatment with ATR inhibitor. For the cell viability assay, 1000 cells of U2OS^ATRX-1^ (−) and U2OS^ATRX-1^ (+) were initially seeded and treated with inhibitor as described above. The same conditions were used for cell viability assays with the U2OS^ATRX-2^ cell line that, for evaluating the ATRX rescued state, was induced with 0.4 μg/ml doxycycline for 7 days.

## Results and Discussion

To evaluate the sensitivity of cell lines to ATR inhibition, we compared the human telomerase positive (“TEL”) cell lines HeLa, HCT116, and MG63 with the ALT cell lines U2OS, CAL72, and SAOS2. Cell viability measurements after treatment with different ATR inhibitor concentrations were conducted using the CellTiter Glo assay (Promega) and by fluorescence-activated cell sorting (FACS) analysis with annexin V staining. The experimental methods and inhibitor treatment conditions of 6 days incubation without medium change were those used previously by Flynn et al. who also studied the MG63, U2OS, CAL72, and SAOS2 osteosarcoma cell lines (3).

### Cell Viability Analysis with the CellTiter Glo Assay

**Figure 1:**
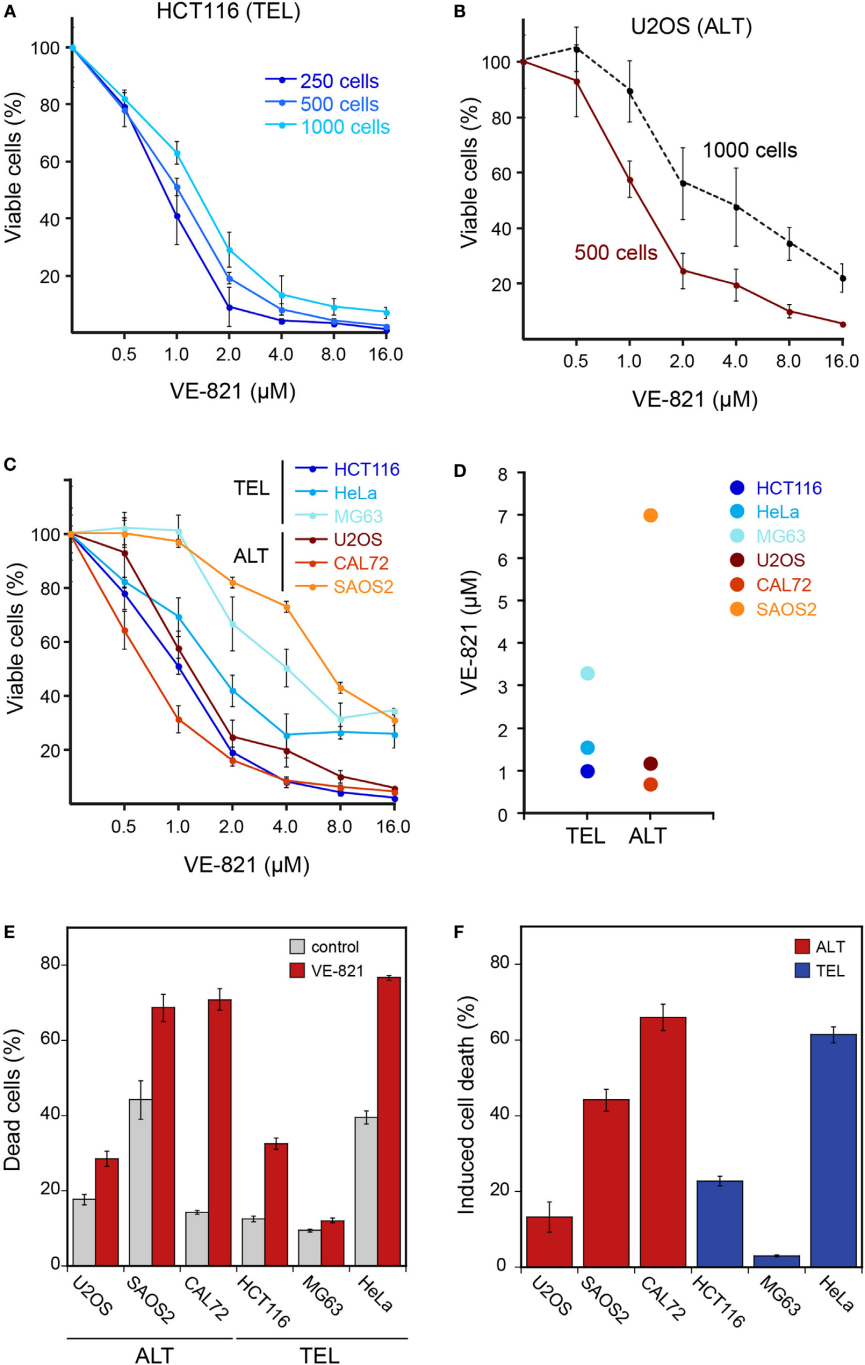
**Cell viability of ALT- and telomerase-positive (TEL) cell lines upon treatment with the ATR inhibitor VE-821**. **(A)** Different cell numbers of HCT116 (TEL) cells were seeded in a 96-well plate and treated with increasing concentrations of the ATR inhibitor VE-821 for 6 days to determine the influence of the starting cell number on the cell viability assay. Cell viability was analyzed using CellTiter Glo and is depicted as percentage of control (*n* = 3). **(B)** Same as panel *A* but for the U2OS ALT cell line. **(C)** Analysis of cell viability for different cell lines with starting cell numbers that led to 70–90% confluency after 6 days in the absence of inhibitor (*n* = 3). Cell numbers used for seeding were 500 cells/well for U2OS, HeLa, HCT116, and MG63 and 1500 cells/well for the CAL72 and SAOS2 cell lines. Error bars represent SD of triplicate experiments. **(D)** IC_50_ concentrations determined from cell viability curves shown in panel *C*. **(E)** FACS analysis of cell death fraction in ALT and TEL cell lines treated with 3 μM VE-821 for 6 days in comparison to the DMSO control. Dead cells were determined by annexin V staining. Error bars represent SEM (*n* = 3). **(F)** Percentage of induced cell death determined from FACS analysis as in panel *E*. The induced cell death fraction was calculated as described in the section “Materials and Methods.”

In order to measure cell viability in response to VE-821, we first examined the HCT116 TEL cell line (Figure 1A) in reference to the original paper that characterized the VE-821 inhibitor (11). It is noted that the CellTiter Glo assay measures changes in the number of viable cells after inhibitor treatment relative to a control sample. Whether the resulting differences originate from a reduced growth rate or from an increase in cell death cannot be distinguished in this assay. The half-maximal inhibitory concentration (IC_50_) for the HCT116 cell line in our experiments was 1–2 μM and similar to that reported previously [see Figure S4D in Ref. (11)]. We note that the results were dependent on the amount of cells initially seeded (Figure 1A). Seeding higher cell numbers shifted the dose–response curve to elevated inhibitor concentrations. Consequently, the cells appeared less sensitive to the inhibitor. The same dependence was observed for the U2OS ALT cell line (Figure 1B). This behavior is likely to reflect the well-known differences between cell lines in terms of their proliferation rate as well as the dependence of this parameter on cell density. For example, cells can appear less sensitive to the ATR inhibitor when starting with higher cell numbers because the proliferating control cells reach confluency and stop dividing before the treatment ends after 6 days. By contrast, cells growing slower upon treatment may not reach confluency within 6 days and continue to proliferate during the complete observation period, albeit at a lower rate. Since values are normalized to the control, this would make the ratio of viable cells in treated versus control samples dependent on the density of seeded cells, the proliferation rate, and the observation time.

### ATR Inhibitor Sensitivity of Different Cell Lines

For comparing ALT and TEL cell lines, we selected a starting cell number for each cell line that led to 70–90% confluency after 6 days in the absence of the inhibitor. The optimized starting cell numbers used for U2OS, HeLa, HCT116, and MG63 were 500 cells, and for the slower growing CAL72 and SAOS2 cell lines 1500 cells/well of a 96-well plate. Using these seeding cell numbers, we found no hypersensitivity of ALT cell lines in response to the VE-821 ATR inhibitor (Figure 1C). Instead, the sensitivity varied between cell lines irrespective of the telomere maintenance mechanism. While the HCT116 TEL and the CAL72 ALT cell lines were sensitive to ATR inhibition at VE-821 concentrations of 1–2 μM, the MG63 TEL and the SAOS2 ALT cell lines showed a stronger reduction of viable cells only at higher inhibitor concentration. The IC_50_ value measured ranged from 0.9 to 3.3 μM for the TEL cell lines and from 0.7 to 7 μM for the ALT cell lines (Figure 1D). Thus, the IC_50_ values of the two groups were not systematically different, but rather showed a high variation within each group.

Next, we quantified dead cells by FACS analysis of annexin V stained telomerase- and ALT-positive cells treated with 3 μM VE-821 for 6 days (Figures 1E,F). Measurements of the SAOS2 ALT cell line and the HeLa TEL cell line revealed a fraction of 40% dead cells already in the control samples in the absence of inhibitor (Figure 1E). This indicates that these cells are more sensitive to 6 days of culturing without medium change. In comparison to the control, the CAL72 ALT cell line displayed the highest sensitivity to ATR inhibitor treatment in this assay, while the MG63 TEL cells were mostly insensitive as reflected by the calculated percentages of induced cell death in relation to the control (Figure 1F). However, treatment with the ATR inhibitor induced a higher percentage of cell death in the HeLa and HCT116 TEL cell lines compared to the U2OS ALT cells. Thus, we did not observe a selective killing of ALT cells by ATR inhibition in these experiments.

### ATR Inhibitor Sensitivity in Dependence of ALT Activity

**Figure 2:**
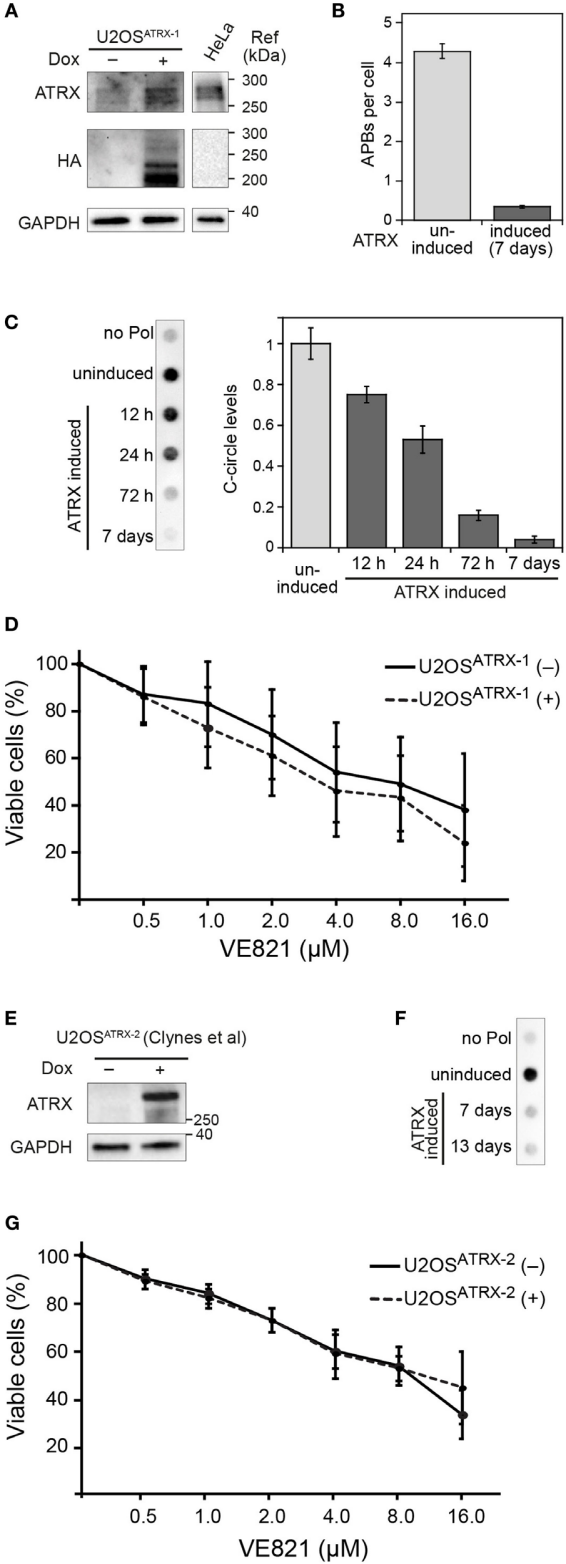
**ALT features and sensitivity to ATR inhibitor treatment upon ectopic expression of ATRX in ATRX-deficient U2OS cells**. **(A)** Western blot showing the expression of HA-tagged ATRX in the generated U2OS^ATRX-1^ cell clone upon doxycycline induction for 48 h and a HeLa reference. In addition to the full-length ATRX band at about 260 kDa, shorter ATRX variants between 200 and 220 kDa were also detected in the HA blot that might correspond to degraded or alternatively spliced products of ATRX, as described previously (15, 16). **(B)** Average number of APBs per cell in uninduced and for 7 days induced U2OS^ATRX-1^ cells (*n* = 350) analyzed by 3D confocal image analysis of PML immunofluorescence and telomere FISH stainings as described previously (10). **(C)** C-circle assay as a marker of ALT activity in uninduced and induced U2OS^ATRX-1^ cells. Samples without polymerase (no pol) and uninduced U2OS^ATRX-1^ were included as controls. The bar plot shows a quantification of C-circle levels in uninduced and induced U2OS^ATRX-1^ cells from three experiments. **(D)** ATR inhibitor sensitivity in dependence of ALT activity in the U2OS^ATRX-1^ cell line. ATRX-induced U2OS^ATRX-1^ (+) and uninduced U2OS^ATRX-1^ (−) cells were analyzed using the CellTiter Glo assay in the presence of increasing concentrations of the ATR inhibitor VE-821 for 6 days. No changes in ATR inhibitor sensitivity were observed when ALT was silenced by ATRX expression. **(E)** Western blot showing the expression of ATRX in the U2OS^ATRX-2^ cell line from (8) after induction for 7 days. **(F)** C-circle assay to test ALT activity in U2OS^ATRX-2^ after 7 and 13 days of doxycycline induction. Samples without polymerase (no pol) and from uninduced cells were included as controls. **(G)** ATR inhibitor sensitivity in U2OS^ATRX-2^ with (-ATRX) and without ALT activity (+ATRX). The viability assay was performed as described in the legend to panel *D*. No changes in ATR inhibitor sensitivity were observed when ALT was silenced by ATRX expression.

To evaluate the effect of ALT activity on ATR sensitivity in an isogenic cell line and independent of the above-mentioned confounding factors, we exploited the recent finding that the α-thalassemia mental retardation X-linked (ATRX) chromatin remodeling protein acts as a suppressor of ALT (8, 12). Accordingly, a cell line for the doxycycline-inducible expression of ectopic ATRX in the U2OS ALT cell line that intrinsically harbors large deletions in the *ATRX* gene was established. In the resulting U2OS^ATRX-1^ cell line, HA-tagged ATRX protein is produced upon induction as confirmed by western blotting using an ATRX- and an HA-specific antibody (Figure 2A). The expression of ATRX protein progressively reduced ALT activity as apparent from monitoring two characteristic ALT markers: single-stranded circular C-rich extrachromosomal telomere repeats (C-circles) as well as PML-telomere colocalizations, termed APBs (13, 14). After 7 days of ATRX expression, the number of APBs was at the background level observed for TEL cell lines (Figure 2B) and C-circles were almost undetectable (Figure 2C) indicating a complete inhibition of ALT activity. Next, we compared the ATR inhibitor sensitivity of U2OS^ATRX-1^ (+) cells that had ALT silenced due to ATRX induction to the same U2OS^ATRX-1^ (−) uninduced cell line with an active ALT pathway. The dose–response curves for the two cell samples were identical as determined with the CellTiter Glo assay (Figure 2D). In order to corroborate these results, we tested another U2OS cell line referred to here as U2OS^ATRX-2^, which was also engineered to express ATRX upon doxycycline treatment and has been demonstrated to suppress ALT upon ATRX induction (8). In line with the data previously published by Clynes et al., 7 days of doxycycline treatment resulted in robust ATRX expression and suppression of the ALT pathway as indicated by the absence of C-circles (Figures 2E,F). Comparing the ATR inhibitor sensitivity of this cell line when ALT was active (no doxycycline) with the same cell line, in which ALT was suppressed (+doxycycline) yielded no differences (Figure 2G). The dose–response curves of the (un)induced U2OS^ATRX-1^ and U2OS^ATRX-2^ cells were indistinguishable within the error of the measurements. Thus, silencing ALT activity via ectopic expression of ATRX did not affect ATR inhibitor sensitivity of the cells.

## Conclusion

Ataxia telangiectasia- and RAD3-related and the protein kinase ataxia telangiectasia mutated (ATM) are the two main DNA damage checkpoint-activating kinases in human cells. Consistent with the view that replication stress and misguided DNA repair synthesis are crucial features of ALT, it was found that inhibition of ATR or ATM decreases ALT activity (3, 10, 17, 18). However, except for the Flynn et al. study, no immediate ALT-specific effects after ATR and/or ATM inhibition on cell viability and proliferation on the time scale of several days have been reported.

In our comparison of different cell lines, we identified a number of factors that affected the apparent sensitivity toward the VE-821 ATR inhibitor but were unrelated to ALT (Figure 1). These included the initial cell number seeded in relation to the proliferation rate as well as differences in the genetic background that may lead to an increased ATR inhibitor sensitivity independent of ALT. For example, the telomerase-positive HCT116 colon cancer cell line used here harbors a mutation in *MRE11*, which impairs binding to NBS1 and Rad50 and suppresses ATM activation in response to replication stress (19, 20). This may account for its relatively high sensitivity toward ATR inhibition in terms of cell viability independent of its telomere maintenance mechanism.

In addition to the effects of the above-mentioned factors, it would still be conceivable that the presence of ALT contributes to an increased sensitivity to ATR inhibition. To address this possibility, we compared two U2OS cell lines in which ALT was active with the same cell line that had ALT silenced by inducing ectopic ATRX expression. In these experiments, the ATR inhibitor sensitivity was not changed when the ALT pathway was rendered inactive (Figure 2). Thus, we conclude that cells that employ ALT to maintain their telomeres are not generally more sensitive to ATR inhibition than telomerase positive cells on the time scale of days. Rather we suggest that, as described above, the cell line-specific genetic background and additional factors exist that are responsible for the different cellular response to ATR inhibition.

Our results indicate that ATR inhibition alone will not be sufficient to target tumors in which ALT is active. Nevertheless, we share the view that the misguided DNA repair and recombination mechanism active in ALT provides unique novel options for anti-cancer therapies. In this context, the recurrent inactivation of the ATRX tumor suppressor protein in ALT cancer samples could be exploited (21). As inactive ATRX is associated with ALT-specific tumor features, it could, for example, be targeted by synthetic lethality approaches. In support of this conclusion, it has been recently shown that ATRX deficiency impairs non-homologous end joining and increases sensitivity to DNA damaging agents in a glioma mouse model (22). A systematic further investigation of this relation appears to be promising for exploiting ALT-associated cellular deregulation in personalized cancer therapies.

## Author Contributions

KD, IC, and CB conducted experiments. KD, IC, and KR designed research and analyzed the data. KR wrote the paper with contributions from KD and IC.

## Conflict of Interest Statement

The authors declare that the research was conducted in the absence of any commercial or financial relationships that could be construed as a potential conflict of interest.
